# Embracing Tuberculosis Preventive Treatment: A Crucial Step towards TB Control in Pakistan

**DOI:** 10.12669/pjms.42.8.20087

**Published:** 2026-08

**Authors:** Anila Basit, Zille Hamayun

**Affiliations:** 1 Anila Basit, Department of Pulmonology, Lady Reading Hospital, Peshawar, Pakistan; 2 Zille Hamayun, Department of Pulmonology, Military Hospital Rawalpindi, Rawalpindi, Pakistan

Tuberculosis (TB), an infectious disease caused by Mycobacterium tuberculosis (MTB), remains a global public health issue throughout the world.^1^ As part of its Global End TB strategy, the World Health Organization has established specific guidelines for the treatment and prevention of TB.^1^ Like other countries, TB also remains a major public health challenge in Pakistan, consistently placing the country among the top five high-burden TB countries in the world, with an estimated 510,000 new cases emerging annually.^2^ As part of the global End TB strategy, Pakistan has also adopted the National Tuberculosis Program (NTP) for the control of TB with multiple targets, including early diagnosis, effective treatment protocols, and engagement of the community to significantly reduce TB incidence and mortality by 2035.^3^ With the aim of 90% reduction in TB related deaths, and 80% reduction in TB incidence, this strategy is the key towards a TB free global society. A key strategy in this program is Tuberculosis Preventive Treatment (TPT), which plays a crucial role in decreasing the incidence of TBI**,** breaking transmission chains and reducing TB-related morbidity.^4^ Latent TBI affects 25% of the world globally and poses a 5-10% risk of progression to TB disease if left un-treated. TBI (TB infection) should be distinguished from TBD (TB disease) by having exposure to, but manifesting no signs, symptoms or tests for TBD.

Despite this, the only hurdle worth mentioning is the inertia of treating physicians. Rest of the hurdles, including drug regimens, difficulties in ensuring patient adherence, and the rising prevalence of primary resistance to isoniazid (INH), are still surmountable.^5^ Overcoming these obstacles is essential to realizing Pakistan’s vision of TB elimination and ensuring the long-term effectiveness of its public health interventions.

TPT implementation is primarily targeted at individuals who are at high risk of developing active TB, including house-hold contacts of confirmed TB cases, people living with HIV/AIDS, and those with immunosuppressive conditions such as those on dialysis, TNF inhibitors, transplant or silicosis.^6^ In addition, high risk population subsets have been identified which include healthcare workers, prisoners, homeless, drug abusers and migrants.

By treating latent TB infection (LTBI), TPT helps reduce the risk of progression to active disease by 60-90%, thereby decreasing the reservoir of potential TB cases. The NTP of Pakistan has prioritized TPT as part of its strategic plan to control TB. To reduce the burden of tuberculosis, one of the main goals is to ensure early detection and treatment of LTBI. This aligns to worldwide TB targets, prioritizing preventive treatment for at least 90% of persons who are eligible by 2025 are in line with this policy.^7^

Different regimens are recommended by The World Health Organization (WHO) for tuberculosis preventive therapy (TPT), each having its distinguish advantages and limitations. Isoniazid (INH) as a monotherapy which is typically administered for 6 to 9 months is one of the most widely used regimens however has several limitations including prolonged treatment duration, poor adherence, and an increased risk of hepatotoxicity. Combined, these often lead to treatment discontinuation, lowering its effectiveness in preventing active tuberculosis.^7^

These issues have been addressed to a great extent with the arrival of newer and short term regimens. Because of its shorter length and better safety profile, rifapentine and INH (3HP) regimen that are taken weekly for three months or daily for one month have demonstrated better adherence. Rifampin monotherapy (4R), a four-month treatment renowned for its outstanding tolerability and adherence rates, is an additional choice.^8^

There are many advantages of tuberculosis preventive therapy (TPT) including reduction in tuberculosis (TB) incidence by proactively treating latent TB infection (LTBI). As a public health intervention, TPT helps suppress the overall burden of TB.^9^ By targeting high-prevalence regions and implementing transmission control strategies, it can significantly improve public health outcomes, making it a crucial public health intervention.

Early intervention can prove to be more cost-effective than managing an active TB case that may require hospitalization, prolonged treatment and additional healthcare resources. An important advantage of TPT is its cost-effectiveness particularly in resource-limited settings like Pakistan, where healthcare systems are already overburdened, and reducing TB-related costs might save resources for other critical health needs.^10^ We have to address concerns regarding drug resistance through appropriate treatment protocols and continuous surveillance of resistance patterns.^9^,^10^

A review of studies examined six RCTs of rifamycin based TPT and found very low risk of bacterial resistance at just 0.01-0.06%: Data published since 1951, 13 studies, IPT = 18095, controls = 17,985. RR of resistance 1.45).^11^ Another landmark systemic review by den Boon et al. (2016) specifically assessed 6 randomized controlled trials of rifamycin-containing TPT regimens and found no significant increase in rifampicin resistance.^12^

Poor documentation of adverse events was reported in a systemic review; however, risk of hepatotoxicity was 2-6% with 6H or 9H treatment regimens. Risk of stopping treatments due to side effects was much lower with 1HP, 3HP regimens. (Module 1: Tuberculosis preventive treatment- WHO2025)

Better adaptation of TPT can be ensured by addressing the concerns of physicians first, and patients later. To make the most of the treatment and minimize its challenges, better monitoring of patients is required to help them adhere to their treatment and improve healthcare systems.

## Striking a Balance: Maximizing Benefits While Mitigating Risks:

To make the most of Tuberculosis Preventive Therapy or TPT while keeping its risks as low as possible we need to take the following steps:

1. We have to keep an eye on drug resistance patterns by regularly checking them which is very important to find and deal with new resistance to Isoniazid or INH.
2. We need to make treatment plans like 3HP more widely available which can help people take their medicine as they should and reduce the risk of adverse side effects.
3. We have to get communities and healthcare providers involved by telling them about the advantages of TPT which can help people accept and follow the treatment.
4. We need to make sure the program is strong by keeping a supply of drugs training healthcare workers and making TPT a part of regular healthcare services, all of which are necessary to meet the targets set by the National Tuberculosis Program or NTP.

Even though TPT has been proven to work it is not used much as it should be in Pakistan because of many problems. Not many healthcare providers know about it the healthcare system is not good enough. People are worried about drugs not working, which has stopped it from being used by many people. However, the good things about TPT are more important than these problems. If we use TPT correctly Pakistan can reduce the number of Tuberculosis cases which will reduce the burden on the healthcare system and make the overall health of the public better. Also, TPT plays an important role in stopping the spread of Tuberculosis that is resistant to drugs, which is a big problem around the world.

To make sure TPT is used successfully Pakistan has to take a few steps. We have to train healthcare providers and make them more aware of TPT so they can find and treat people who’re eligible for it. We need to improve our ability to diagnose Tuberculosis and make TPT services more available so we can find latent Tuberculosis infections or LTBI on time and start treatment. Moreover, we have to set up systems to monitor and evaluate the treatment so we can track whether people are taking their medicine find potential problems and measure the effect of TPT programs. Finally, we have to address the concerns about drug resistance by using the right treatment protocols and continuously monitoring them which will help keep the first-line Tuberculosis treatment effective while stopping the spread of strains. By taking these measures, Pakistan can get the most out of TPT. Come closer, to achieving its goals of eliminating Tuberculosis.

Tuberculosis preventive treatment (TPT) in Pakistan presents a tough challenge in the country’s fight against TB. While on one hand, it is a powerful public health tool that can significantly reduce transmission and protect vulnerable populations, and at the same time ensure its implementation which is complex and demands careful evaluation of potential risks, including adherence issues, drug-related side effects, and programmatic constraints.

While TPT offers real promise in curbing the TB epidemic, its success depends on thoughtful execution and continuous monitoring to minimize unintended harm. Reaching the ambitious targets set by the National TB Program (NTP) will require coordinated efforts from policymakers, healthcare providers, and communities alike. Embracing TPT as a key strategy, Let us prioritize TPT and work together towards a TB-free Pakistan.
